# Hypothermic Shock Applied After Perinatal Asphyxia Prevents Retinal Damage in Rats

**DOI:** 10.3389/fphar.2021.651599

**Published:** 2021-04-08

**Authors:** Manuel Rey-Funes, Daniela S. Contartese, Rafael Peláez, Josune García-Sanmartín, Judit Narro-Íñiguez, Manuel Soliño, Juan Carlos Fernández, Aníbal Sarotto, Nicolás S. Ciranna, Juan José López-Costa, Verónica B. Dorfman, Ignacio M. Larrayoz, C. Fabián Loidl, Alfredo Martínez

**Affiliations:** ^1^ Laboratorio de Neuropatología Experimental, Instituto de Biología Celular y Neurociencia “Prof, E. De Robertis” (IBCN), Facultad de Medicina, CONICET - Universidad de Buenos Aires, Buenos Aires, Argentina; ^2^ Departamento de Biología Celular, Histología, Embriología y Genética, Instituto de Biología Celular y Neurociencia “Prof, E. De Robertis” (IBCN), Facultad de Medicina, Universidad de Buenos Aires, Buenos Aires, Argentina; ^3^ Biomarkers and Molecular Signaling Group, Center for Biomedical Research of La Rioja, Logroño, Spain; ^4^ Angiogenesis Group, Oncology Area, Center for Biomedical Research of La Rioja, Logroño, Spain; ^5^ Centro de Estudios Biomédicos Básicos, Aplicados y Desarrollo, Universidad Maimónides, Buenos Aires, Argentina

**Keywords:** hypothermia, perinatal asphyxia, apoptosis, electroretinogram, cold-shock proteins

## Abstract

Perinatal asphyxia (PA) can cause retinopathy and different degrees of visual loss, including total blindness. In a rat model of PA, we have previously shown a protective effect of hypothermia on the retina when applied simultaneously with the hypoxic insult. In the present work, we evaluated the possible protective effect of hypothermia on the retina of PA rats when applied immediately after delivery. Four experimental groups were studied: Rats born naturally as controls (CTL), animals that were exposed to PA for 20 min at 37°C (PA), animals exposed to PA for 20 min at 15°C (HYP), and animals that were exposed to PA for 20 min at 37°C and, immediately after birth, kept for 15 min at 8°C (HYP-PA). To evaluate the integrity of the visual pathway, animals were subjected to electroretinography at 45 days of age. Molecular (real time PCR) and histological (immunohistochemistry, immunofluorescence, TUNEL assay) techniques were applied to the eyes of all experimental groups collected at 6, 12, 24, and 48 h, and 6 days after birth. PA resulted in a significant reduction in the amplitude of the a- and b-wave and oscillatory potentials (OP) of the electroretinogram. All animals treated with hypothermia had a significant correction of the a-wave and OP, but the b-wave was fully corrected in the HYP group but only partially in the HYP-PA group. The number of TUNEL-positive cells increased sharply in the ganglion cell layer of the PA animals and this increase was significantly prevented by both hypothermia treatments. Expression of the cold-shock proteins, cold-inducible RNA binding protein (CIRP) and RNA binding motif protein 3 (RBM3), was undetectable in retinas of the CTL and PA groups, but they were highly expressed in ganglion neurons and cells of the inner nuclear layer of the HYP and HYP-PA groups. In conclusion, our results suggest that a post-partum hypothermic shock could represent a useful and affordable method to prevent asphyxia-related vision disabling sequelae.

## Introduction

The most severe complication in perinatology services across the world is perinatal asphyxia (PA) (Lee et al., 2013; Cunningham et al., 2018; Rego et al., 2018). PA induces a global and transient lack of oxygen supply to the fetus immediately previous, during, or immediately after birth. Clinically, and in a fashion independent from its severity, PA may generate either no deleterious effects, result in the death of the infant, or produce different degrees of short and/or long-term central nervous system (CNS) damage (Volpe 1987). The most common sequelae resulting from PA are attention deficit hyperactivity disorder, epilepsy, intellectual disability, spasticity, and visual or auditory alterations (Hill 1991; Younkin 1992; Crofts et al., 1998; Herrera et al., 2018; Albrecht et al., 2019; Workineh et al., 2020).

For the last 30 years, our group has been using a PA rat model that closely recapitulates the human condition. With this model we have described, in the surviving animals, lesions in several areas of the CNS, such as the brain, the spinal cord, and the visual system (Loidl et al., 1994; Dorfman et al., 2009). In the retina, asphyctic newborns develop severe long-term neurological injuries, which include several degrees of PA-induced retinopathy (Rey-Funes et al., 2010). The changes observed in the retina of asphyctic animals include ganglion cell death, neovascularization, and Müller cell hypertrophy in the inner retina (Rey-Funes et al., 2013). The nitrergic system is one of the factors involved in the genesis of all these lesions (Rey-Funes et al., 2011; Rey-Funes et al., 2016).

When PA occurs at low temperature, perinatal mortality is avoided and the CNS is protected from damage (Loidl et al., 1997; Loidl et al., 1998). This response involves a decrease in nervous tissue metabolism, which reduces the formation of reactive oxygen and nitrogen species, thus inhibiting the toxicity caused by these free radicals (Ekimova 2003; Gisselsson et al., 2005; Rodrigo et al., 2005; Rey-Funes et al., 2011).

Based on the results obtained in previous animal experimentation, the use of hypothermia is currently the standard of care to treat episodes of severe PA-ischemia in humans (World Health Organization, 1991; Thoresen, 2015; Rivero-Arias et al., 2019). Nevertheless, most clinical studies still focus on the positive effects of hypothermia in the brain while studies on the beneficial effects on the visual system are still scarce (Akin et al., 2019; Grego et al., 2020).

For a long time, it was postulated that the beneficial effects of hypothermia were mainly due to decreased metabolism including lower enzymatic activity, but additional molecular mechanisms underlying the beneficial effects of hypothermia have been discovered in the last few years (Al-Fageeh and Smales 2006). Whereas most proteins reduce their expression when exposed to cold temperatures, there is a small family of proteins whose expression increases under these conditions. These proteins are known as cold-shock proteins and include cold-inducible RNA binding protein (CIRP) and RNA binding motif protein 3 (RBM3) (Chip et al., 2011; Tong et al., 2013). These proteins, which belong to the heterogeneous nuclear ribonucleoprotein family, bind to cellular RNAs and regulate their half-life and thus their expression potential and final functions (Lleonart 2010; Wellmann et al., 2010; Liu et al., 2013). These cold-shock proteins have been identified in the mammalian brain (Schmitt et al., 2007; Tong et al., 2013) and retina (Larrayoz et al., 2016).

Therapeutic hypothermia is typically applied over long periods (days) (Azzopardi et al., 2008), which may produce undesired side effects, but we have demonstrated that a short exposure to the cold (hypothermic shock, 15–20 min) is enough for inducing expression of the cold-shock proteins, at least in newborns (Larrayoz et al., 2016).

In previous articles, we have widely shown that reducing temperature during PA prevents retinal damage (Rey-Funes et al., 2010; Rey-Funes et al. 2011; Rey-Funes et al. 2013) but whether postnatal application of hypothermia could protect from vision loss was not known. Therefore, to evaluate a clinically feasible way of applying hypothermia, in this study we have compared the effects of applying hypothermia either during or after PA in the prevention of retinal alterations. The morphological and physiological manifestations of PA in the retina were studied, paying special attention to the potential role of CIRP and RBM3 in this process.

## Materials and Methods

### Ethics statement

Animals were cared for in accordance with the guidelines of the NIH *Guide for the Care and Use of Laboratory Animals* (National Research Council 2011). Sprague-Dawley albino rats with genetic quality and sanitary certification from the animal facility of our institution were cared for in accordance with the guidelines published in the ARVO Statement for the Use of Animals in Ophthalmic and Vision Research. Treatments described below were approved by the Ethical Committee of CICUAL (Comité Institucional para el Uso y Cuidado de Animales de Laboratorio, Resolution Nº 3,021/2019), Facultad de Medicina, Universidad de Buenos Aires, Argentina. Appropriate proceedings were performed to minimize the number of animals used and their suffering, pain, and discomfort. Animals were kept under standard laboratory conditions, with light/dark cycles of 12/12 h, and food and water were given *ad libitum*.

### Perinatal asphyxia Model

**FIGURE 1:**
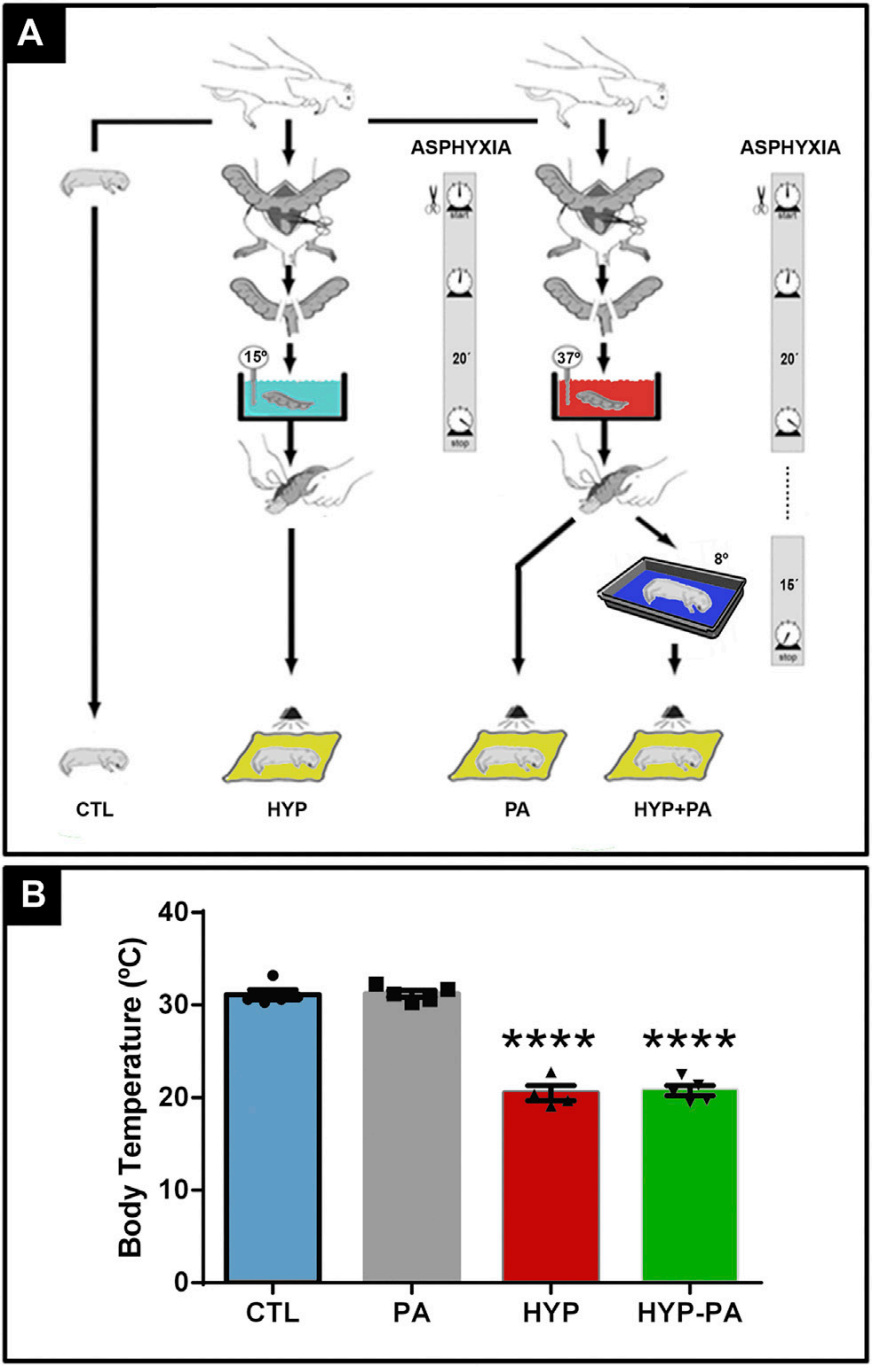
Experimental model design and hypothermic treatment. **(A)**: Severe perinatal asphyxia was induced by a hypoxia-ischemia non-invasive model. Term pregnant rats were sacrificed and immediately hysterectomized after the first pup was delivered vaginally. These normally delivered pups were used as controls (CTL). The remaining full-term fetuses, still inside the uterine horns, were transiently immersed in a water bath for 20 min at 37°C (PA) or at 15°C (HYP) for asphyxia induction. After asphyxia, the uterine horns were opened, pups were removed, and placed for recovery under a heating lamp for 1 h. Alternatively, after PA treatment fetuses were removed from the uterine horns and immediately exposed to 8°C for 15 min (HYP-PA). **(B)**: Core temperature of pups was measured using a digital thermometer probe. Significant decrease of core temperature in HYP and HYP-PA related to CTL and PA normothermic groups confirmed the efficacy of both cold treatments. Two way ANOVA, significant interaction F = 164.2, *p* < 0.0001, Tukey´s multiple comparison test, *p* < 0.0001 (CTL vs. HYP and PA vs. HYP-PA). Asterisks indicate significant differences with CTL. ****: *p* < 0.0001.

Severe PA was induced using a noninvasive model of hypoxia-ischemia, as previously described (Loidl et al., 1998), with slight modifications. Term pregnant rats were sacrificed by decapitation and immediately hysterectomized after their first pup was delivered vaginally. These normally delivered pups were used as controls (CTL group). The remaining full-term fetuses, still inside the uterine horns, were subjected to asphyxia performed by transient immersion in a water bath at 37°C for 20 min (PA group), or at 15°C for 20 min (HYP group). After asphyxia, the uterine horns were opened, pups were removed, dried of their fluids, and their umbilical cords were ligated. Alternatively, following perinatal asphyxia at 37°C, some fetuses were removed from the uterine horns, dried up, and immediately exposed to 8°C for 15 min in a temperature controlled room (HYP-PA group). All pups were placed for recovery under a heating lamp for 1 h and those fulfilling the inclusion criteria (occipitocaudal length >41 mm, weight >5 g, healthy respiratory frequency, good motility, vocalization, and healthy skin color) were given to surrogate mothers. Each surrogate mother received between 8 and 10 pups and all were successfully brought to weaning age (Figure 1A). To avoid the influence of hormonal variations due to the female estrous cycle, only male pups were studied. Male pups were identified by the larger distance between the genital tubercle and the anus. In order to confirm the efficiency of the hypothermic treatment, core temperature was measured using a digital thermometer (TES-1300, TES Electrical Electronic Corp. Taipei, Taiwan) connected to a K type thermocouple (TPK-01). The temperature and duration of the post-partum cold shock were chosen from our previous study where we showed that 15 min at 8°C was enough to induce cold shock proteins in rat pups (Larrayoz et al., 2016).

### Tissue Collection

Animals were sacrificed at different time points, according to the requirements of each technique. For histology assays, including immunohistochemistry, immunofluorescence, and TUNEL, animals were deeply anesthetized with 300 mg/kg ketamine (Imalgene, Merial Laboratorios, Barcelona, Spain) plus 30 mg/kg xylazine (Xilagesic, Proyma Ganadera, Ciudad Real, Spain), eyes enucleated, fixed in 4% paraformaldehyde in 0.1 M pH 7.4 phosphate buffer at 4°C for 48 h, dehydrated, and paraffin embedded. For mRNA amplification and Western blotting, animals were decapitated, eyes enucleated, and kept frozen at −80°C.

### TUNEL Assay

Enucleated eyes of 6-day-old rats (n = 5 animals per group) were paraffin-embedded. Serial sections (5 μm thick) were obtained with a microtome (Leica, Wetzlar, Germany) and mounted onto coated slides. Tissue sections were probed for apoptosis-associated DNA fragmentation by terminal deoxynucleotidyl transferase dUTP nick end labeling (TUNEL) with the *In Situ* Cell Death Detection POD Kit (Roche, Basel, Switzerland), following manufacturer´s instructions. In order to confirm negative results, TUNEL-processed sections were incubated with 10 IU/ml DNaseII (Sigma Chemical Co.) in 50 mM Tris–HCL, pH 7.5, 10 mM Mg_2_Cl, and 1 mg/ml BSA, for 10 min at room temperature. An additional negative control was done by omitting the deoxynucleotidyl transferase enzyme.

### Immunohistochemistry and Multiple Immunofluorescence

Fixed enucleated eyes of 24-h-old rats (n = 5 animals per group) were paraffin-embedded. Tissue sections (5 µm-thick) were dewaxed, rehydrated through graded ethanol, and incubated with a blocking solution containing 10% normal serum in PBS (pH 7.4) for 1 h. Immunoreactivity was detected by incubating slides overnight at room temperature with a single primary antibody (Table 1). Immunoreactivity was detected with biotinylated goat anti-rabbit (PK-6101) or anti-mouse (PK-6102) IgG, followed by incubation with avidin-biotin complex (ABC Vectastain Elite kit, Vector Laboratories, Burlingame, California, USA), as appropriate. The specificity of the assays was corroborated in adjacent sections by omission of the primary antibodies. The reaction was visualized with 3,3′diaminobenzidine (DAB) intensified with nickel ammonium sulfate (SK-4100, DAB kit, Vector Laboratories), yielding a gray/black product. Bright field microscope images were captured with an optic microscope (BX40, Olympus Optical Corporation, Tokyo, Japan), fitted with a digital camera (390CU 3.2 Megapixel CCD Camera, Micrometrics, Spain).

**TABLE 1:** Primary and secondary antibodies used in this study.

Primary antibodies				
*Target*	*Species*	*Dilution*	*Source*	*References*
CIRP	Mouse monoclonal	1:300	Proteintech	60025-2-Ig
RBM3	Rabbit polyclonal	1:1,000	Proteintech	14363-1-AP

Secondary antibodies				
*Specificity*	*Fluorochrome*	*Dilution*	*Source*	*References*
Donkey anti rabbit	Alexa fluor 555	1:200	Molecular probes	A31572
Donkey anti mouse	Alexa fluor 488	1:200	Molecular probes	A21202

For co-localization studies, a mixture of both anti-CIRP and anti-RBM3 antibodies were used in an overnight incubation at 4°C, exposed to the proper secondary antibodies (Table 1), and counterstained with DAPI (1:1,000, Sigma). The specificity of the assay was corroborated in adjacent sections by omission of the primary antibodies. Images were acquired using a confocal microscope (Nikon C1 Plus Laser microscope, Nikon Corp. Tokyo, Japan) and were analyzed with the EZ-C1 software (v3.9, Nikon Ltd. London, United Kingdom). To corroborate the specificity of the immunodetection, sequential line scanning (lambda strobing mode) was used to eliminate any crosstalk emission between the fluorophores. Control sections using each single antibody were also scanned by the three lasers to verify that emission was detected only in the specific single channel.

### Image analysis

Care was taken on selecting anatomically matched areas of retina among animals before assays. Fields were chosen in the central region of retinal cross-sections and the number of positive cells in ten ×40-objective fields was recorded. To avoid variations in the determination of the specific immunoreactivity and in the quantification process, all the images for the same marker were obtained the same day and under the same light and contrast intensity. Only those cells that had a gray level darker than a defined threshold criteria (defined as the optic density 3-fold higher than the mean background density) were considered as specific immunoreactive cells. The background density was measured in a region devoid of immunoreactivity, immediately adjacent to the analyzed region. To evaluate the relative abundance of CIRP- or RBM3-immunoreactive and TUNEL-positive cells, those cells marked by pixels that exceed the defined threshold density were counted. The image software Micrometrics SE P4 (Standard Edition Premium 4, Micrometrics, Spain) was used. Adobe Photoshop software (Adobe Photoshop CS5, Adobe Systems Inc. Ottawa, ON, Canada) was used for digital manipulation of brightness and contrast when preparing the figures.

### Electroretinograms

Fortyfive-day-old rats (n = 10 per experimental group) were subjected to electroretinography, as described (Rey-Funes et al., 2017). Briefly, after overnight adaptation in the dark, rats were anesthetized under dim red illumination. An ophthalmic solution of 5% phenylephrine hydrochloride and 0.5% tropicamide (Fotorretin, Poen, Buenos Aires, *Argentina*) was used to dilate the pupils. Rats were placed facing the stimulus at a distance of 25 cm in a highly reflective environment. A reference electrode was placed through the ear, a grounding electrode was attached to the tail, and a gold electrode was placed in contact with the central cornea. Scotopic electroretinograms (ERG) were recorded from both eyes simultaneously and 20 responses were collected to flashes of unattenuated white light (1 ms, 1 Hz) from a photic stimulator (light-emitting diodes) set at maximum brightness. The registered response was amplified (9 cd s/m^2^ without filter), filtered (1.5-Hz low-pass filter, 500 Hz high-pass filter, notch activated), and averaged (Akonic BIO-PC, Buenos Aires, *Argentina*). The a-wave was measured as the difference in amplitude between the recording at onset and the trough of the negative deflection and the b-wave amplitude was measured from the trough of the a-wave to the peak of the b-wave. Values from each eye were averaged, and the resultant mean value was used to compute the group means a- and b-wave amplitudes ±SEM. To calculate oscillatory potentials (OP), the same photic stimulator was used with filters of high (300 Hz) and low (100 Hz) frequency. The amplitudes of the OP were estimated by using the peak-to-trough method. The sum of four OP was used for statistical analysis.

### RNA Isolation and Quantitative Polymerase Chain Reaction (qRT-PCR)

Posterior chambers of the eyes of 6-, 12-, 24-, and 48-h-old neonatal rats (n = 6 per group) were homogenized with TRIzol (Invitrogen, Madrid, Spain) and RNA was isolated with the RNeasy Mini kit including a DNase I on-column digestion (Qiagen, Germantown, MD). One µg of total RNA was reverse-transcribed into first-strand cDNA using random primers and the SuperScript III kit (Invitrogen) in a total volume of 20 μL according to the manufacturer’s instructions. Reverse transcriptase was omitted in control reactions to confirm lack of contamination from genomic DNA. Resulting cDNA was mixed with SYBR Green PCR Master Mix (Applied Biosystems, Carlsbad, CA) for quantitative real time polymerase chain reaction (qRT-PCR) using 0.3 µM forward and reverse oligonucleotide primers for CIRP and RBM3 (Table 2). Quantitative measures were performed using a 7,300 Real Time PCR System (Applied Biosystems). Cycling conditions were an initial denaturation at 95°C for 10 min, followed by 40 cycles of 95°C for 15 s, 60°C for 1 min, and 72°C for 30 s. At the end, a dissociation curve was implemented from 60 to 95°C to validate amplicon specificity. Gene expression was calculated using relative quantification by interpolation into a standard curve. All values were relativized to the expression of the house keeping gene 18 S.

**TABLE 2:** Primers used for qRT-PCR.

Primer (target)	Sequence	Expected product size
CIRP-forward	GCA​TCA​GAT​GAA​GGC​AAG​GT	64 bp
CIRP-reverse	CCAGCGCCTGCTCATTG	
RBM3-forward	TGG​AGA​GTC​CCT​GGA​TGG​G	65 bp
RBM3-reverse	TGG​TTC​CCC​TGG​CAG​ACT​T	
18 S-forward	ATG​CTC​TTA​GCT​GAG​TGT​CCC​G	101 bp
18 S-reverse	ATT​CCT​AGC​TGC​GGT​ATC​CAG​G	

### Statistical analysis

All data were analyzed with GraphPad Prism 8.0 software and were considered statistically significant when *p* < 0.05. Values are expressed as means ± standard error of the mean (SEM). Normally distributed data were evaluated by one way ANOVA followed by the Dunnet’s (Bonferroni) post-hoc test while data not following a normal distribution were analyzed with the Kruskal-Wallis test followed by Dunn´s test.

## Results

### A Short Hypothermic Shock Is Enough to Reduce Core Body Temperature

Four experimental groups have been used in this study: Rats born naturally that were used as controls (CTL), animals that were exposed to PA for 20 min at 37°C (PA), animals exposed to PA at 15°C (HYP), and animals that were exposed to PA at 37°C and, after birth, were kept for 15 min at 8°C (HYP-PA). After recovery, all pups were given to surrogate mothers to complete postnatal development (Figure 1A).

At the time of birth, pups belonging to the CTL and PA groups had a rectal temperature of 31.3 ± 1.3 and 30.8 ± 0.8°C, respectively. When PA was induced under hypothermia, rectal temperature decreased to 20.6 ± 1.0°C. Hypothermic exposure after asphyxia, in which the neonates were placed for 15 min in a cold room at 8°C, also decreased rectal temperature to 20.6 ± 1.0°C (Figure 1B).

### Hypothermia prevents Perinatal Asphyxia-Induced Changes in the Electroretinogram

**FIGURE 2:**
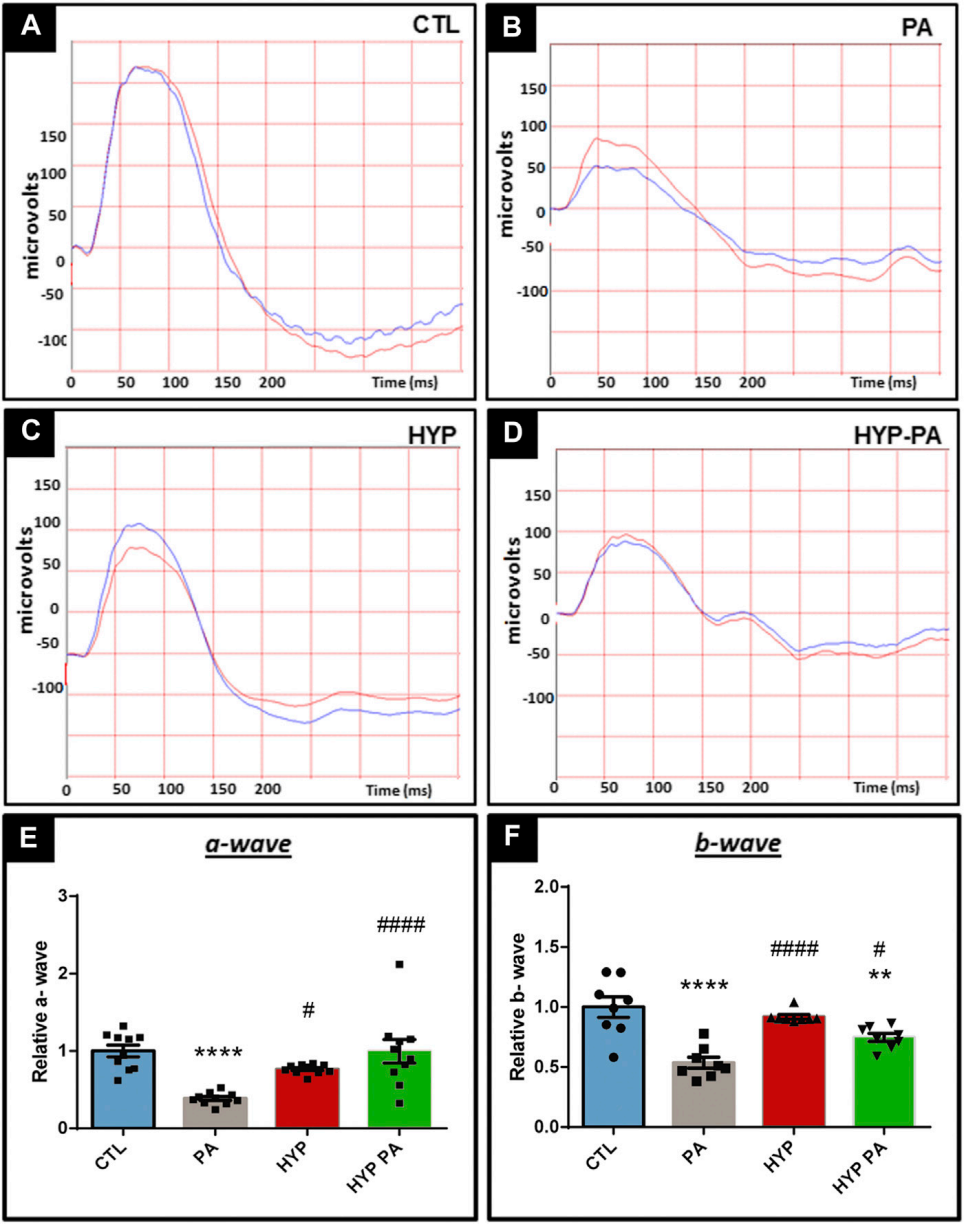
Hypothermia prevents changes in the electroretinogram induced by perinatal asphyxia. Representative electroretinograms of 45 day-old animals (n = 10 per group) subjected to PA with and without hypothermic shock. **(A)**: Control (CTL), **(B)**: Normothermic perinatal asphyxia (PA), **(C)**: Hypothermia during perinatal asphyxia (HYP), and **(D)**: Hypothermic shock after perinatal asphyxia (HYP-PA). **(E)**: Amplitude of the a-wave in the four experimental groups. Perinatal asphyxia (PA) induced a significant decrease in the a-wave with respect to controls (CTL), whereas both hypothermic treatments (HYP and HYP-PA) prevented it. Two way ANOVA, significant interaction F = 11.14, *p* < 0.0001, Tukey´s multiple comparison test, *p* < 0.0001 (CTL vs. PA), *p* = 0.019 (PA vs. HYP), *p* < 0.0001 (PA vs. HYP-PA). **(F)**: Amplitude of the b-wave in the four experimental groups. Perinatal asphyxia (PA) induced a significant decrease in the b-wave compared to controls (CTL), whereas both hypothermic treatments (HYP and HYP-PA) prevented it. Two way ANOVA, significant interaction F = 15.62, *p* < 0.0001, Tukey´s multiple comparison test, *p* < 0.0001 (CTL vs. PA), *p* = 0.0095 (CTL vs. HYP-PA), *p* < 0.0001 (PA vs. HYP), *p* = 0.036 (PA vs. HYP-PA). Each bar represents the mean ± SEM of 10 animals. Asterisks indicate significant differences with CTL. **:*p* < 0.01; ****:*p* < 0.0001. Pound signs indicate significant differences with PA. #:*p* < 0.05; ####:*p* < 0.0001.

**FIGURE 3:**
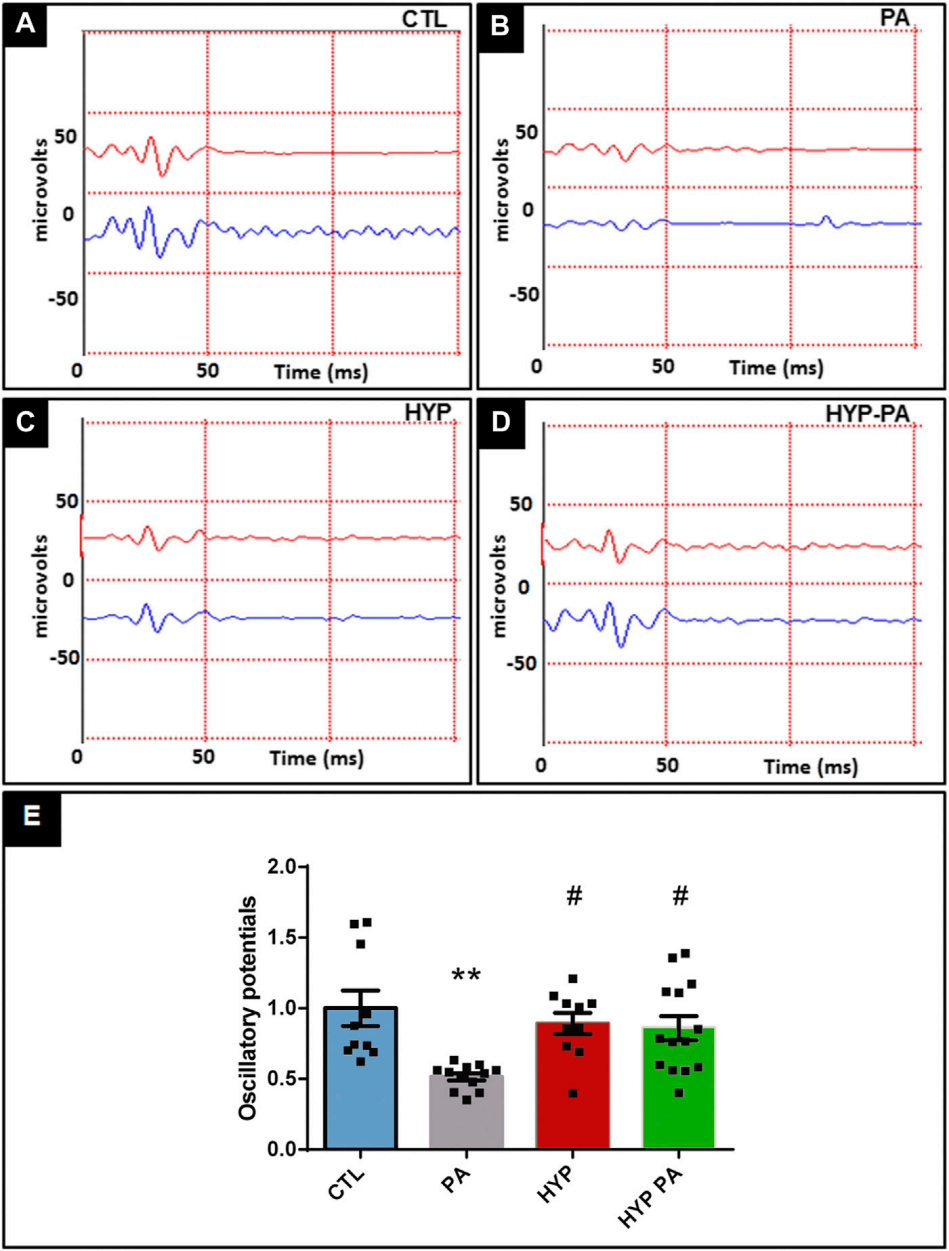
Hypothermia prevents changes in the oscillatory potentials induced by perinatal asphyxia. Representative graphics of oscilatory potentials of 45 day-old animals (n = 10 per group) subjected to PA in normothermic or hypothermic conditions. **(A)**: Control (CTL), **(B)**: Normothermic perinatal asphyxia (PA), **(C)**: Hypothermia during perinatal asphyxia (HYP), **(D)**: Hypothermic shock after perinatal asphyxia (HYP-PA). **E**: Quantification of the amplitude of the OP. Perinatal asphyxia (PA) induced a significant decrease in the amplitude of the OP compared to the control group (CTL), whereas both hypothermic treatments (HYP and HYP-PA) prevented it. Two way ANOVA, significant interaction F = 6.37, *p* = 0.0012, Tukey´s multiple comparison test, *p* = 0.0012 (CTL vs. PA), *p* = 0.016 (PA vs. HYP), *p* = 0.017 (PA vs. HYP-PA). Each bar represents the mean ± SEM of at least 10 animals. Asterisks indicate significant differences with CTL. **:*p* < 0.01. Pound signs indicate significant differences with PA. #:*p* < 0.05.

Electroretinograms performed 45 days after birth showed that PA animals had a significant decrease in the amplitude of the a-wave, b-wave, and oscillatory potentials (OP) when compared to the CTL group (Figures 2A,B, 3A,B). All parameters in the HYP group were indistinguishable from the CTL group (Figures 2C,E,F, 3C,E). In contrast, the HYP-PA group had the same a-wave and OP amplitude as CTL group (Figures 2D,E, 3D,E) but only partially recovered b-wave amplitude (Figures 2D,F).

### Perinatal Asphyxia-Induced Apoptosis Was Prevented by Hypothermia During or After Asphyxia

**FIGURE 4:**
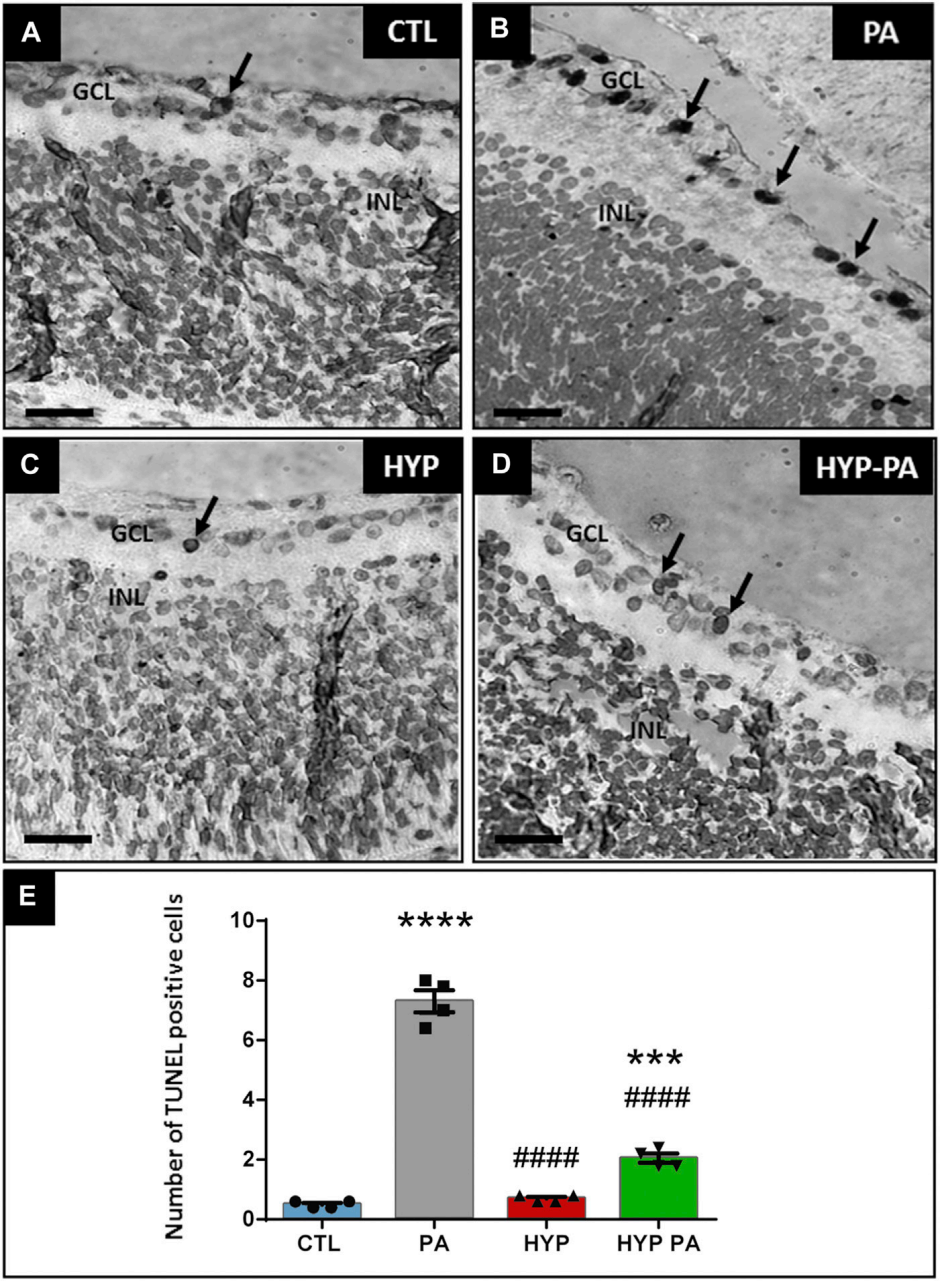
Apoptosis induced by perinatal asphyxia is prevented by hypothermia. Representative images of apoptotic cells (arrows) localized in the ganglion cell layer as labeled by the TUNEL assay in the four experimental groups: control (CTL, **(A)**), normothermic perinatal asphyxia (PA, **(B)**), hypothermia during perinatal asphyxia (HYP, **(C)**), and hypothermia after perinatal asphyxia (HYP-PA, **(D)**). GCL: ganglion cell layer. INL: inner nuclear layer. Scale bars: 40 µm. **(E)**: Graphical representation of TUNEL-positive cell number. PA group showed a significant increment in the number of TUNEL positive cells compared to CTL group. Hypothermia (HYP and HYP-PA groups) significantly prevented apoptosis induction. Two way ANOVA, significant interaction F = 244.5, *p* < 0.0001, Tukey´s multiple comparison test, *p* < 0.0001 (CTL vs. PA), *p* < 0.001 (CTL vs. HYP-PA), *p* < 0.0001 (PA vs. HYP and PA vs. HYP-PA), *p* = 0.0025 (HYP vs. HYP-PA). Each bar represents the mean ± SEM of 5 animals. Asterisks indicate significant differences with CTL. ***: *p* < 0.001; ****:*p* < 0.0001. Pound signs indicate significant differences with PA. ####:*p* < 0.0001.

Perinatal asphyxia induced a significant increase in the number of TUNEL-positive apoptotic cells in the ganglion cell layer at 6 days after birth (Figure 4B) when compared to CTL (Figure 4A). The number of apoptotic cells was 7-fold higher in PA animals when compared to the CTL group (Figure 4E). This PA-induced increase was completely prevented in those animals that were subjected to hypothermia during perinatal asphyxia (HYP) (Figures 4C,E). The hypothermic treatment applied after perinatal asphyxia (HYP-PA) partially prevented retinal apoptosis (Figures 4D,E).

### RBM3 Expression Is Induced by Hypothermia During or After Asphyxia

**FIGURE 5:**
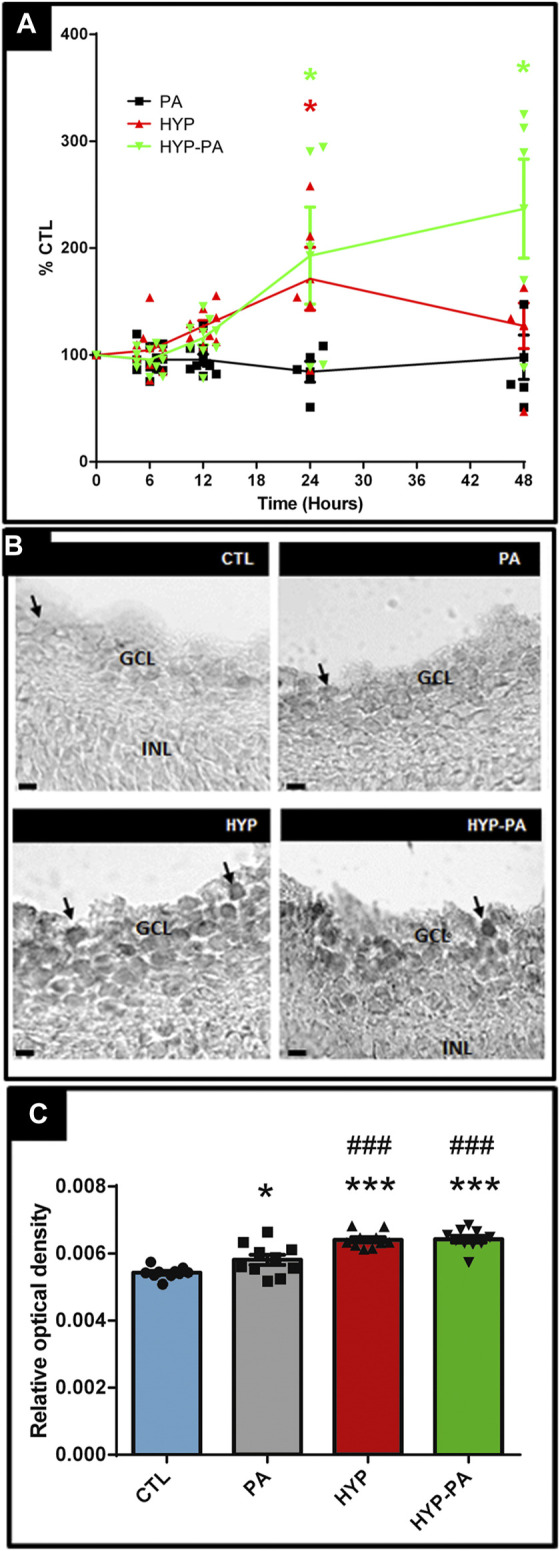
RBM3 expression is elevated by hypothermia. **(A)**: Relative RBM3 mRNA expression through time as measured by qRT-PCR. A significant increment of RBM3 mRNA expression was induced by both cold treatments (HYP and HYP-PA groups) compared to CTL and PA groups at 24 h after perinatal asphyxia. However, 48 h after asphyxia, RBM3 expression remained significantly elevated only in the HYP-PA group. All values were relativized to CTL values (n = 6 animals per group). Two way ANOVA, significant interaction F = 7.05, *p* < 0.0001, Tukey´s multiple comparison test, *p* = 0.0022 (CTL vs. HYP, 24 h), *p* < 0.0001 (CTL vs HYP-PA and HYP vs. HYP-PA, 24 h), *p* < 0.0001 (all groups vs. HYP-PA, 48 h). Asterisks indicate time points where a significant difference with CTL was found. **(B)**: Representative images of RBM3 immunoreactive cells (arrows) in the ganglion cell layer of control (CTL), normothermic perinatal asphyxia (PA), hypothermia during perinatal asphyxia (HYP), and hypothermic shock after perinatal asphyxia (HYP-PA) animals. INL: Inner nuclear layer, GCL: ganglion cell layer. Scale bars: 10 µm. **(C)**: Relative optical density of RBM3-positive cells. A significant increment was shown in the number of RBM3-positive cells in 24 h old HYP and HYP-PA when compared to CTL and PA groups (n = 5 per group). Two way ANOVA, significant interaction F = 23.40, *p* < 0.0001, Tukey´s multiple comparison test, *p* = 0.049 (CTL vs. PA), *p* < 0.0001 (CTL vs. HYP and CTL vs. HYP-PA), *p* < 0.001 (PA vs. HYP and PA vs. HYP-PA). Asterisks indicate significant differences with CTL. *: *p* < 0.05; ****: *p* < 0.0001. Pound signs indicate significant differences with PA. ###:*p* < 0.001.

RBM3 mRNA expression showed significant induction in those groups exposed to cold treatments (HYP and HYP-PA groups) compared to CTL and PA groups (Figure 5A). A time-course evaluation of RBM3 mRNA expression showed strong induction by hypothermia at 24 h after cold treatment (Figure 5A). Forty-eight hours after birth, only the RBM3 mRNA expression of HYP-PA animals remained significantly elevated with respect to the other groups (Figure 5A). This difference in RBM3 mRNA expression was in agreement with a significant increment in the number of RBM3-immunoreactive cells in 24-h old HYP and HYP-PA animals as compared to CTL and PA groups (Figures 5B,C). There was a significant increase in staining intensity in the PA group (Figures 5B,C), indicating that asphyxia may also induce RBM3 expression.

### CIRP Expression Is Induced by Hypothermia During or After Asphyxia

**FIGURE 6:**
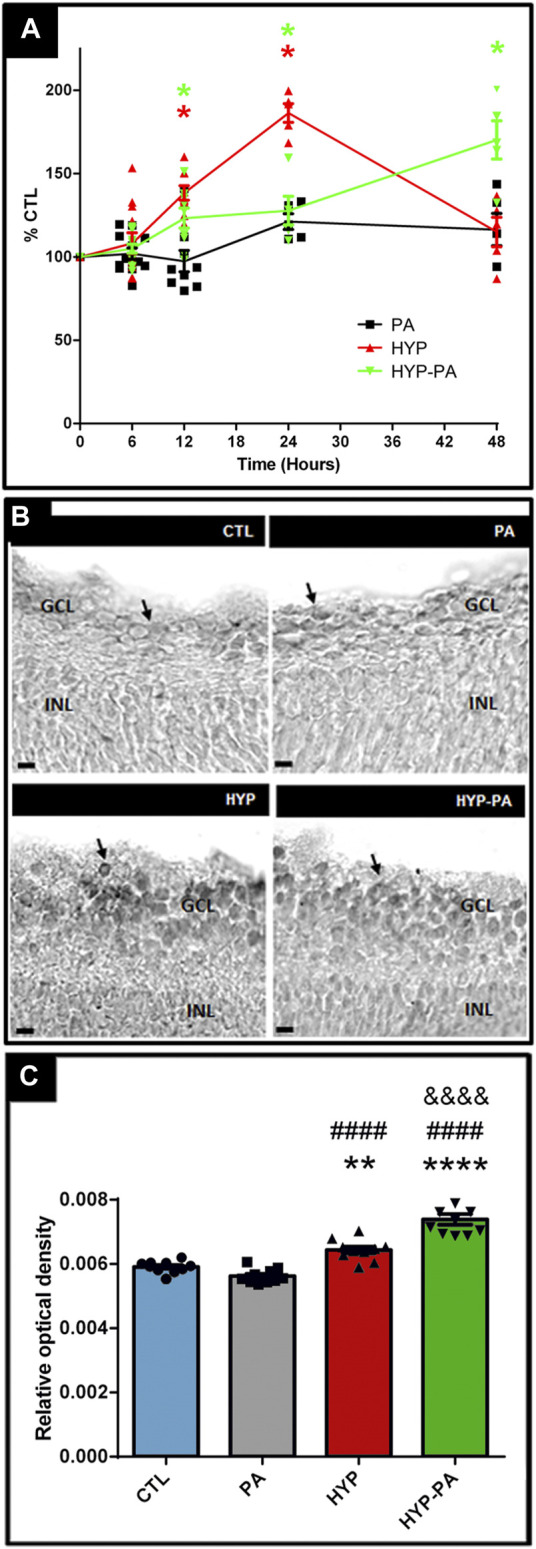
CIRP expression is induced by hypothermia. **(A)**: Relative CIRP mRNA expression through time as measured by qRT-PCR. The time-course expression of CIRP showed significant changes among treatments from 12 to 48 h after asphyxia. Significant induction of CIRP mRNA expression was determined in all groups compared to CTL, however 48 h after perinatal asphyxia, CIRP expression remained significantly increased only in the HYP-PA animals. All values were relativized to CTL values (n = 6 animals per group). Two way ANOVA, significant interaction F = 12.37, *p* < 0.0001, Tukey´s multiple comparison test, *p* < 0.0001 (CTL vs. HYP, 12 h), *p* = 0.0058 (CTL vs. HYP-PA, 12 h), *p* < 0.0001 (CTL vs. HYP and HYP vs. HYP-PA, 24 h), *p* = 0.018 (CTL vs. HYP-PA, 24 h), *p* < 0.0001 (all groups vs. HYP-PA, 48 h). Asterisks indicate time points where a significant difference with CTL was found. **(B)**: Representative images of CIRP immunoreactive cells (arrows) in the ganglion cell layer of control (CTL), normothermic perinatal asphyxia (PA), hypothermia during perinatal asphyxia (HYP), and hypothermic shock after perinatal asphyxia (HYP-PA) animals. INL: Inner nuclear layer, GCL: ganglion cell layer. Scale bars: 10 µm. **(C)**: Relative optical density of CIRP-positive cells. A significant increment in the number of CIRP-immunoreactive cells in HYP and HYP-PA when compared to CTL and PA groups was detected (n = 5 per group). Two way ANOVA, significant interaction F = 50.88, *p* < 0.0001, Tukey´s multiple comparison test, *p* = 0.0082 (CTL vs. HYP), *p* < 0.0001 (CTL vs. HYP-PA), *p* < 0.0001 (PA vs. HYP and PA vs. HYP-PA), *p* < 0.0001 (HYP vs. HYP-PA). Asterisks indicate significant differences with CTL. **: *p* < 0.01, ****: *p* < 0.0001. Pound signs indicate significant differences with PA. ####: *p* < 0.0001. Ampersand signs indicate significant differences with HYP. &&&&: *p* < 0.0001.

CIRP mRNA expression showed modulation by hypothermia (Figure 6A). A time-course evaluation of CIRP mRNA expression showed a significant induction by hypothermia 12 and 24 h after birth with both cold treatments (HYP and HYP-PA groups) (Figure 6A). Interestingly, at 48 h, the CIRP mRNA expression values for the HYP group were undistinguishable from those of the CTL and PA groups, whereas the expression in the HYP-PA group was significantly increased (Figure 6A). This difference in CIRP mRNA expression correlated with a significant increment in the number of CIRP-immunoreactive cells in HYP and HYP-PA animals as compared to CTL and PA groups (Figures 6B,C).

### RBM3 and CIRP Co-localization Related to Hypothermic Treatment

**FIGURE 7:**
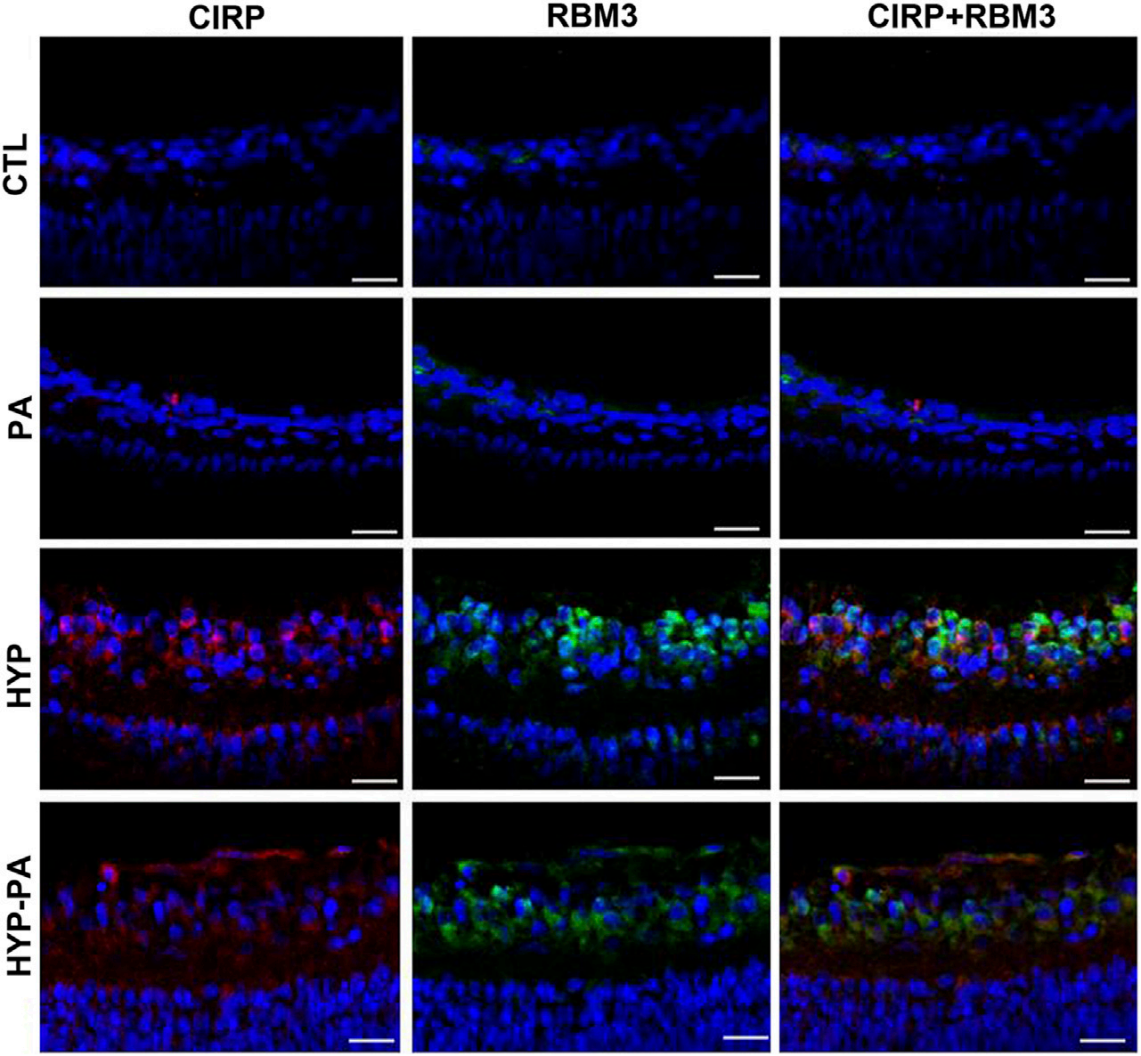
Colocalization of RBM3 and CIRP in cold-treated retinas. The expression of RBM3 (green) and CIRP (red) was very low in the retinas of CTL **(upper row)** and PA **(second row)** animals. The expression of both proteins was highly induced following exposure to hypothermia either during (HYP, **third row)** or after (HYP-PA, **bottom row)** perinatal asphyxia. A broad colocalization pattern was evident for both proteins in the cytoplasm of ganglion cells. Cell nuclei were counterstained with DAPI (blue). Tissues were collected at 24 h after birth. Scale bars: 10 µm.

Immunoreactivity for both cold-induced proteins, RBM3 and CIRP, was not detectable in the retinas of animals not exposed to hypothermia (CTL, PA) but showed an intense expression in the inner layers of the retina of hypothermic animals (HYP and HYP-PA) at 24 h after birth, with a cytoplasmic distribution. Both proteins showed some degree of co-localization in the same cells (ganglion cells and some cells of the INL) (Figure 7).

## Discussion

In this study, we have confirmed that PA induces intense disturbances in electroretinogram patterns and causes extensive apoptosis of ganglion cells in the inner retina, supporting the concept that PA produces serious damage to the integrity of the visual system. Interestingly, a short exposure to therapeutic hypothermia, either during or after asphyxia, was able to significantly correct both parameters. This indicates that hypothermia may prevent visual dysfunctions.

Measurement of core temperature following hypothermia showed that all pups were cooled to around 20°C (a drop of approximately 12°C from their regular temperature) irrespective of the time of application (during or after the asphyxia). This indicates that applying cold to neonates is tantamount to cooling the fetuses during perinatal asphyxia, which obviously is impractical in the clinic and is a purely experimental paradigm. All previously obtained data using hypothermia during perinatal asphyxia (Loidl et al., 1997; Loidl et al., 2000; Rey-Funes et al., 2011; Rey-Funes et al., 2013) could be almost directly applied to postnatal cooling, which is a treatment already used in the clinic to prevent brain complications (Edwards et al., 2010; Rutherford et al., 2010).

Electroretinography methods showed that PA has a very disruptive effect on the pattern of the a-wave, b-wave, and OP. Similar results were previously obtained using the same model of PA (Fernandez et al., 2020). It is understood that the a-wave represents the response of the photoreceptor cells, the OP that of the cells located in the inner nuclear layer, and the b-wave that of the ganglion cells (Jung et al., 2015; Matei et al., 2020; Zhai et al., 2020), thus indicating that PA imposes a negative influence at all levels of the visual axis. Interestingly, our study shows that application of hypothermia, either during or after PA, completely restored the a-wave and OP in all animals. On the other hand, the b-wave was only partially restored by application of hypothermia after birth (HYP-PA), suggesting that this technique may not completely prevent all damage to the retina. The lack of complete recovery of the b-wave in HYP-PA animals may be due to the higher number of apoptoses found in the retinas of these rats in comparison with the HYP group.

The observation of TUNEL-positive ganglion cells in the retina of rats exposed to PA indicates a massive destruction of the intraretinal optical pathway induced by PA, which would be responsible for severe vision losses. The number of TUNEL-positive cells was significantly lower in the animals treated with hypothermia than in those suffering PA. Even though the protective effect was more remarkable when the cold shock was applied during PA, it was also highly beneficial when provided post-partum.

The use of therapeutic hypothermia has been intensified in newborns with hypoxic-ischemic encephalopathy applying exclusively whole body cooling (Edwards et al., 2010) or combined with cephalic cooling at 33.5°C for 72 h (Azzopardi et al., 2008). Therapeutic hypothermia managed to decrease brain tissue damage and improve survival and neurological outcomes up to 18 months of age (Edwards et al., 2010; Rutherford et al., 2010). When applied globally, hypothermia produces a significant decrease in body metabolism at a general level. In addition, it involves a series of events that regulate the transcription, transport, and organization of the cytoskeleton, cell cycle, gene expression, and various cellular metabolic processes (Safar and Kochanek 2002; Sonna et al., 2002; Hoyoux et al., 2004). Among the processes that are activated during cold exposure, cold-inducible proteins, including CIRP and RBM3, were identified in human cells (Chip et al., 2011; Tong et al., 2013). These proteins regulate gene expression by binding the 5′-or 3′-regions of specific mRNAs and modulating the translation and stability of their transcripts. The binding motif sequences for CIRP and RBM3 have been identified (Lleonart 2010; Xia et al., 2012) and are present in the mRNAs of some factors such as VEGF (vascular endothelial growth factor), PEDF (growth factor derived from the pigment epithelium), and MMP9 (matrix metalloproteinase 9), among others. These proteins have been shown to participate actively in the regulation of PA-induced deleterious changes to the retina, which include angiogenesis and gliosis (Fernandez et al., 2020), and could be possible molecular targets of action for the cold-inducible proteins. In the present study, we have determined that a brief exposure to cold temperature induces the expression of RBM3 and CIRP in the retina, showing a similar pattern for both proteins. In addition, the expression of these proteins was similar whether the hypothermic stimulus was applied either during or after PA.

Recent reviews have linked the experimental data obtained in animals and the potential applications of hypothermia for preventing neural damage, including retinal injury, in human newborns (Albrecht et al., 2019; Xi 2020). Interestingly, many native cultures around the world practice dipping newborns in cold water soon after birth (Connell-Szasz 1988; Degefie et al., 2014; Sacks et al., 2015). Although the current reasons to perform this rite are rather vague, it may constitute a manifestation of traditional wisdom based on age-old observations of the beneficial consequences of hypothermia for the health of the newborn. In that regard, it would be easy to convince new mothers and their families to accept this treatment based on their own folk lore and ancient traditions.

In conclusion, exposure to hypothermia post-partum induces the expression of cold-inducible proteins CIRP and RBM3 in the retina, and reduces morphological and physiological manifestations of retinal damage. Given the brief therapeutic shock studied in this work, this method may be beneficial when compared with the potential side effects of prolonged exposures to cold. The present results obtained in rats support initiating clinical trials to evaluate whether a short hypothermic shock may be helpful in preventing retinal damage in asphictic neonates.

## Data Availability

The raw data supporting the conclusions of this article will be made available by the authors, without undue reservation.
